# The weight of school grades: Evidence of biased teachers’ evaluations against overweight students in Germany

**DOI:** 10.1371/journal.pone.0245972

**Published:** 2021-02-08

**Authors:** Mona Dian, Moris Triventi

**Affiliations:** 1 Department of International Business Administration, University of Applied Sciences Worms, Worms, Rhineland-Palatinate, Germany; 2 Department of Sociology and Social Research, University of Trento, Trento, Trentino-Alto Adige, Italy; Waikato Institute of Technology, NEW ZEALAND

## Abstract

Discrimination and prejudice against overweight people is common in Western societies. In this article we aim to understand whether these attitudes reverberate into the school setting, by investigating whether teachers grade overweight students more severely than comparable normal weight students. By relying on the Attribution-Value Model of Prejudice (AVMP) and previous studies, we test a series of hypotheses using data from the German National Educational Panel Study (NEPS SC3) on a sample of students enrolled in the 7^th^ grade (lower secondary education). We used hierarchical ordered logit regression to assess whether overweight and obese students receive systematically lower grades by their teachers in German and mathematics, adjusting for subject-specific competences measured with a standardized test, and a rich set of socio-demographic and socio-psychological students’ characteristics (e.g. the “big five”). Results suggested that overweight and obese students were more severely graded in both subjects. The penalty for overweight students, and especially for obese students, was slightly larger in German and in the lowest part of the grade distribution. There was also indication of heterogeneous penalties by gender, with overweight male students being especially penalized in math. Possible ways to help teachers in assigning grades in a fairer way are discussed at the end.

## Introduction

Discrimination against overweight people in the labor market and in social life is well documented and increased over time [1, 2]. In the labor market, overweight people are less likely to be hired [3] and are at risk to receive a lower starting salary in comparison to normal weight people [4]. In social life, overweight people are more often bullied [5] and receive less friendship requests in social networks on the internet [6].

In this article we aim to understand whether obese and overweight people constitute stigmatized and penalized social categories in a specific context that has attracted comparatively less attention, namely the school. In particular, we are interested in how teachers evaluate the scholastic performance of overweight students compared to normal weight students in German lower secondary school.

In most previous studies, school grades are mainly intended as indicators of academic proficiency, assuming that body mass diminishes performance in school via poor health and a resulting reduction in study effectiveness [7]. Nonetheless, this perspective overlooks the fact that overweight is a socially sanctioned feature of the physical body [8] and that lower grades may result from social pathways involving discrimination and stigma by teachers [9].

The literature on teachers’ grading in school posits that grades are not always a fair measure of subject-specific competences because they can also reflect teachers’ attitudes about the students [10, 11]. As for all common people, teachers’ attitudes are likely to be influenced by attributes that are not relevant to cognitive outcomes such as student gender [12], migration background [13], and body size [9]. These attributes might elicit stereotypes about the students’ competence and thus lead to discrimination in grading [12–14].

While gender and migration background are commonly considered to be unchangeable, overweight is perceived as a controllable attribute that is culturally undesirable at the same time [15]. The Attribution-Value Model of Prejudice (AVMP) argues that attributions of controllability and cultural value of the attribute the stereotype concerns are required to be simultaneously present to lead to prejudice. When weight is believed to be controlled volitionally, overweight people can be held responsible for their situation and be treated as if they deserved the blame [16]. But overweight is only considered a negative attribute if there is a cultural preference for thinness. Without the negative cultural value people would not be blamed for overweight [15, 17].

It fits to the controllability aspect of the AVMP that overweight people are perceived as lacking personal control, being lazy, less conscientious [18, 19], and less disciplined [20]. As control and discipline are important for competence development [21], stereotypes about overweight people also include stupidity [18], slowness in thinking [22], lower competence and sloppiness [19]. In comparison to prejudice against other groups, negative attitudes about overweight people are widespread and expressed more openly. Social desirability does not hold opinions back as much as in the case of racism or homophobia [23, 24]. Indeed, a large part of the population displays prejudice against overweight people referring to their intelligence and willingness to perform [18, 23, 25]. Some studies suggest that also school teachers might have prejudice against overweight people [26, 27].

The cultural preference for thinness in Western countries is also well documented. In all socioeconomically highly developed societies thin bodies have the highest ratings of attractiveness [28]. Weight loss up to thinness or underweight is the aim of many women who are uncomfortable with their body [29]. Therefore, there is ample evidence suggesting that attributions of controllability and negative cultural value are met in Western societies, especially regarding women’s bodies. A likely result from prejudiced attitudes and beliefs is discriminatory behavior [4], and indeed, overweight students receive lower grades than their peers [30, 31].

Against this background, our research questions are: Are overweight and obese students graded more severely by their teachers than equally competent normal weight students? Moreover, does the potential grading bias vary by subject and gender? The answers to these questions are relevant for at least two reasons.

The first reason is fairness for the sake of the effect of grades on students’ motivation to learn and on their well-being [32]. Students who felt treated unfairly by their teachers are more stressed and are more likely to have psychological problems [33]. The second reason is fairness for the sake of the validity of grades in signaling competence and mastery of knowledge in specific subjects [34]. Grades are relevant signals in educational-related decisions [35, 36] and thereafter in application processes, e.g. when future employers screen potential job applicants and when the university admits new students [10, 37]. Grades of overweight students can result from a mix of prejudice and objective ratings of competence, leading to the underestimation of their academic proficiency and potential for skill development. If this is the case, employers and institutions risk to unintentionally disregard qualified employees.

We investigate the potential grading bias by using data of the German National Educational Panel Study with students attending the seventh grade [38]. By this way, we aim to contribute to the literature by using recent high-quality nationally representative data on lower secondary education in Germany. The German context differs from the US context in two ways. First, German students are placed in educational tracks already in secondary education [see 35 for more information on the German tracking system]. Teachers’ grades are particularly important for students’ track placement, which in turn is crucial for later educational transitions. Second, while in the US obesity is comparatively widespread (37.3% in 2016), the incidence of this phenomenon in Germany is lower (25.7% in 2016) [see 39 for prevalence rates]. It could be that obesity is a less visible characteristic in the German context, leading to a stronger stigma as the society may be less aware of discrimination against overweight people [40].

The paper is organized in the following way: We set the basis for the research hypotheses by outlining results from the previous literature about grading bias towards overweight people. After presenting the German National Educational Panel Study data, variables and our methods, we report the empirical findings of the hierarchical ordinal logistic regression models. The article concludes with a discussion of the results and possible implications for teachers and schools.

### Teachers, students’ weight and grading

In the school context, the beliefs of teachers about students’ competences and work ethics matter and overweight students face difficulties that can have consequences for their future careers. In a study of Neumark-Sztainer and colleagues [26], half of the school staff thought that overweight was self-caused. About one fifth believed that overweight people were less tidy and less successful at work. Fitting to the prejudicial attitudes, the teachers’ perceptions of competences in math and reading decrease with increasing BMI of the students [41]. Teachers say that overweight students are burdensome to have in the classroom [27] and, for this reason, might judge them more severely than normal weight students.

Some studies reveal a relationship between student BMI and school grades: After controlling for sociodemographic factors, overweight students have a lower grade point average than normal weight students [42], a lower average grade in the main subjects [30], and are less likely to report having mostly higher grades [31]. However, these studies do not necessarily prove teacher bias as they did not control for student level of subject-specific competence in the statistical models. Therefore, the lower grades possibly express a lower competence of overweight compared to normal weight students, as suggested by a few studies about brain and memory functioning [43–45]. However, only a very low number of studies find a connection between overweight and lower competence measured by standardized test scores in a real-world setting [42, 46]. Mostly, competences do not differ between overweight and normal weight students after adjusting for sociodemographic characteristics [47–50].

Other studies, using American samples, go further and partly account for competences as an alternative cause of grade differences to discrimination. Sabia [51] reports an association between a higher BMI and a lower grade point average controlling for general intelligence in a sample of 14 to 17 years old white females. But holding general intelligence constant may not rule out differences in subject-specific competences as students also need to have enough discipline and motivation to study [52]. MacCann and Roberts [53], controlling for competence in math and vocabulary, found a lower average grade in various school subjects among obese students compared to normal weight students in the US. Competence scores for science and social studies were, however, not included and could have still biased measures of grade differences. Research of this quality is not available for countries with a smaller share of overweight people in the population.

Despite some heterogeneity in the research findings, most works found that BMI is negatively related to academic achievement. Moreover, several studies report that teachers might have prejudice against overweight students. Following these insights, and regarding our research question if overweight and obese students are graded more severely, we expect that

*overweight and obese students get lower grades than normal weight students*, *once adjusting for subject-specific competence and other individual characteristics*

(Hyp. 1).

The second research question asked whether teachers’ bias varies by subject. In this work, we focus on two key subjects in secondary education, namely German and mathematics. If discrimination against overweight students is just an inherent feature of the teachers in their role of graders, we could predict a similar degree of bias in both subjects. If instead the way in which students are commonly tested and the standards usually adopted by the teachers play a role as well, we could expect some differences in the grading bias across subjects. In mathematics, most exams are based on exercises with a precise solution, whereas in German teachers assess students more often with oral exams and open-ended questions, in which subjective elements have more room to affect the evaluation process. If these characteristics matter, we should expect that

overweight and obese students are graded less generously than comparable normal weight students especially in German and less in mathematics

(Hyp. 2).

Our third research question asks whether teachers’ grading bias differs between males and females. It has been argued that obesity reduces the perception of femininity by others but not as much as it reduces the perception of masculinity [54]. In subjects related to reading and writing activities, for example English, femininity is a favorable attribute, whereas in mathematics it is often considered an unfavorable attribute. Obese girls are regarded less feminine and have a smaller femininity advantage in English classes than other girls. Consequently, in comparison to normal-weight girls, it is likely they face a specific penalization [9]. Indeed, previous studies in the US found that obese girls face higher grading penalization compared to normal-weight girls in English rather than in mathematics. For boys, no association between obesity and academic performance was found at all [9]. Following this logic, we hypothesize that

the weight penalty regarding grades is higher among girls than among boys, especially in German

(Hyp. 3).

### Analytical design

#### Data

We make use of the German National Educational Panel Study (NEPS)–Starting Cohort 3, which provides information on students attending the grade 7 in 2012, who are repeatedly interviewed in the subsequent years [38]. The longitudinal data set is used in a cross-sectional manner by analyzing data from wave 3, because the variables of interest are not measured repeatedly in every wave. Education- and school-related variables as well as competences and socio-demographics are assessed via paper-and-pencil interviews (PAPI) or provided by official student lists. Information on the parents is collected using computer-assisted telephone interviews (CATI) [55, 56].

We restricted the sample by excluding students without matches in the parent and institution data set. 47% of the cases from the original target data set were deleted because they had no matches (institution or parent data). After this operation, we explored the data and found that while the incidence of missing values is null or modest for several socio-demographic variables, the share of missing values is higher for the outcomes (around 7%) and especially for the main independent variable (25%) (see S1 Table). If we had performed a complete information case analysis, we would end up with 2,142 students. Since relying on listwise deletion would lead to underpowered and possibly biased estimates, we applied a multiple imputation (MI) procedure to impute the missing values [57], which allowed us to rely on an analytical sample of 3,754 cases. Under the assumption of random missing values conditional on the covariates, MI is able to minimize the bias, maximize the use of available information and obtain appropriate estimates of uncertainty around the point estimates [58, 59]. To impute the missing values, we followed the canonical three steps:

1. Imputation phase: We filled the missing data with estimated values and created a complete dataset using the chained equations/MICE approach, which uses a separate conditional distribution for each imputed variable [60]. We relied on multinomial logit to model student BMI, ordinal logit to model teacher grades and parental educational level (ISCED), a linear OLS to model students’ test scores, parental occupational status (ISEI), psychological traits (“big five”), and the indexes measuring the attachment to school and homework duration. The other variables (age, gender, native language and school region, type of secondary school attended) do not present any missing value and therefore are used in the imputation models as additional predictors. Considering the amount of missing values and following recommendations from recent literature [61], we generated 50 imputations (completed datasets) under our chosen imputation model.
2. Analysis Phase: We applied the hierarchical ordinal logistic regression models (see below) to analyze teachers’ grading in each of the 50 complete data sets.
3. Pooling Phase: The parameter estimates (coefficients and standard errors) obtained from each analyzed data set are then combined for inference using the so-called ‘Rubin’s rules’, which take into account properly the variation between imputations [57, 58].

#### Variables

The variables come from several sub-data sets of the NEPS. The sub-data sets contain information from the student lists, the students and parents, and standardized competence tests. In the case of students, all time-variant variables were measured in the third wave while the time-invariant variables (gender, native language) were recorded in the first wave. The information on parents is measured once in the first wave and updated in subsequent waves in case of changes.

The grades in mathematics and German are self-reported by the students. Taking into consideration the highly skewed distribution of the original variable and to maintain enough statistical power for the analysis, we recoded the original grades into four categories: *low* (poor, failing, and passing), *medium-low* (satisfactory), *medium-high* (good) and *high* (very good). Alternative but reasonable classifications lead to substantially similar results to the ones presented here.

Body mass index (BMI) categories were built using the BMI-for-age growth charts of the National Center for Health Statistics [62]. The charts provide BMI percentiles for children grouped by gender and age in months, measured in the middle of the months. We assigned the BMI percentiles to children with the respective age in months rounded down. The BMI is calculated using the formula weight divided by height in meters squared [63]. Height and weight were self-reported by the students. Biologically implausible values of weight, height, and BMI were excluded using an external reference table [64]. Following previous contributions [63], the variable BMI is classified in four qualitatively distinct weight types: 1) Underweight (lower than the 5th percentile); 2) Normal weight (BMI greater than or equal to the 5th percentile and lower than the 85th percentile); 3) Overweight (higher than or equal to the 85th percentile but lower than the 95th percentile); 4) Obese (higher than or equal to the 95th percentile). Even though the literature highlights that BMI is not a perfect measure of body fatness since it does not account for the ways in which body composition differs by individuals’ characteristics such as muscularity, sex, and age [65], these problems are less marked in our context. Indeed, the growth charts we used are age- and gender-specific; moreover, muscularity should not be a big problem regarding children younger than 16 years old.

We control for subject-specific competence as an alternative cause of lower grades instead of teacher bias. Subject-specific competence is measured by weighted maximum likelihood estimates (WLE), which are corrected for the position of the test domain in the test book. The resulting score is the best estimate of each respondent’s observed answers to several items belonging to a domain [66, 67]. The domains are mathematics and reading competence. A negative WLE score signals a below-average competence, a score of 0 an average competence, and a positive score an above-average competence.

Aside from competences, other variables need to be accounted for because they can influence both grades and weight. Parents’ socioeconomic status (SES) is negatively associated to overweight [68] and positively related to grades [69]. Children with a higher conscientiousness show less risky health-related behavior [70] and have higher grades [71]. In general, personality traits are linked to over- and underweight [72] as well as to academic achievement [73]. Furthermore, migration background can be a confounder, since children with a migration origin are more often overweight than other children [74] and they also have a lower academic performance [13]. We control for native language as a proxy for migration background, which is not available in the data. Lastly, attitudes about learning and objective behaviors could be the reason why overweight students receive lower grades. To rule out that they put less effort in learning activities than normal-weight students, we control for attitudes using attachment to school and objective behaviors using homework duration. The complete list of control variables and their description is presented in Table 1.

**Table 1:** Characteristics of the control variables.

Variable	Item from NEPS	Type of Answer
Reading competence (test scores)	Booklet with 29/30 reading competence items	WLE score corrected for the test position, computed by the NEPS staff
Mathematics competence (test scores)	Booklet with 23 mathematics competence items	WLE score corrected for the test position, computed by the NEPS staff
Gender	School’s list: gender child	0—female
		1—male
School region	Please enter the postal code of your school.	1—former West Germany
		2—former East Germany incl. Berlin
Age in years	When were you born?	Open question
Native language	Now let’s talk about your mother tongue: which language did you learn as a child in your family?	0—German only
		1—other native language
Parental SES	International Socio-Economic Index of occupational status (ISEI) (Highest most recent ISEI among pairs, most recent ISEI if single parent)	16 to 90
Parental ISCED	International Standard Classification of Education (ISCED) (Highest most recent ISCED among pairs, most recent ISCED if single parent)	1–2 or lower
		2—3b
		3—3a & 3c
		4—4a & 5b
		5—5a & 6
School type	Sample: type of school	0—Hauptschule
		1—Realschule
		2—Gymnasium
		3—schools with different tracks
Openness	I do not care much about arts. [R] & I have an active imagination, I am an imaginative person.	9 step sum scale from 2 items: 1—does not apply at all *to* 5—applies completely
Conscientiousness	I am easy-going and tend to be a bit lazy. [R] & I am thorough.	
Extraversion	I tend to be cautious, reserved. [R] & I am out-going and sociable.	
Agreeableness	I tend to be critical of other people. [R] & I trust other people easily, I believe in the goodness in people.	
Neuroticism	I am relaxed and don’t get easily stressed. [R] & I am considerate, sensitive.	
Attachment to school	Going to school for a long time is a waste of time.	1—completely disagree *to*
		5—completely agree
Homework duration	How much time do you normally spend on your homework and learning for school?	1—less than half an hour per day
		2—about half an hour to 1 hour per day
		3—about 1 to 2 hours per day
		4—about 2 to 3 hours per day
		5—about 3 to 4 hours per day
		6—more than 4 hours per day

## Methods

To identify teachers’ grading bias against overweight and obese students with observational data we apply a so-called “grade-equation” approach [75] in which teachers’ marks are expressed as a function of the categories of body mass index (BMI), adjusted for students’ subject-specific competences measured with a standardized test (TESTSCORES), and an additional set of covariates capturing students’ heterogeneity in socio-demographic characteristics (SOCIODEMO), attitudes (ATTITUDES) and behavior (BEHAV), which may be correlated with BMI and with teachers’ grades as well. With this strategy it is possible to assess to what extent overweight and obese students receive on average different marks compared to their most similar normal weight peers, in terms of demonstrated performance in a specific subject and other relevant traits. This strategy assumes that standardized test scores are a less subjective measure of individual school-related competences than teachers’ grades and could work as a yardstick to assess whether and how much factors other than student ability are taken into account by teachers when attributing grades to students [76]. For each school subject we estimated two model specifications, which can be succinctly described as follows:

Model1:GRADESj=f(BMI,TESTSCORE,SOCIODEMO)

Model2:GRADESj=f(BMI,TESTSCORE,SOCIODEMO,ATTITUDES,BEHAV)

Model 1 is intended to measure the total teacher grading bias, which we can interpret as the average deviation of teachers’ evaluations from a more objective and standardized form of competence assessment: According to the models, if students with comparable competence scores receive different grades, the grade difference identifies teachers’ bias. Model 2 includes possible mediators that can explain the total teacher grading bias. If the included mediators are sufficient to capture all the possible relevant mechanisms by which teachers’ judgements are distorted, then we can interpret the coefficients corresponding to the BMI variable as the result of (taste or statistical) discrimination processes [76].

In the last part of the analysis, we investigate whether the grading bias due to overweight and obesity is heterogeneous across boys and girls, by introducing an interaction term between gender (SEX) and BMI. For the sake of simplicity, we omitted the main effects of the interacted variables in the notation, which are included in all models to avoid unbiased estimates, as suggested by Brambor et al. [77].

Model3:GRADESj=f(BMI×SEX,TESTSCORE,SOCIODEMO,ATTITUDES,BEHAV)

Given the nature of our outcome variables and the data/sampling structure, we use three-level ordinal hierarchical (random-intercept) regression models in which individuals are nested in courses that are nested in schools. By this way we allow the standard errors to be dependent within classes and schools, taking into account that students within the clusters are more similar to each other than between the clusters, resulting from exposure to similar contexts. The statistical models are estimated separately for German and mathematics, and in each model, the subject-specific test score is included as a control variable. Accounting for potential non-linearities in the relationship between the competence variables and the outcomes does not alter the results in a significant way. As described above, each model was estimated on each of the 50 datasets generated from our imputation model and then combined in order to take into account the variability between the estimates across the imputed data.

## Results

### Descriptive statistics and bivariate analysis

The analytical sample is constituted by 51% of males and 20% of the students speak other native languages beneath German. Among the types of secondary schools, 61% were enrolled in the Gymnasium (high-level track), 20% in the Realschule (medium-level track), 5% in the Hauptschule (lowest-level track), and 14% in schools with different tracks (comprehensive). Regarding the BMI, 80% (n = 1719) of the students have a normal weight, 11% (n = 230) have underweight, 7% (n = 149) have overweight, and 2% (n = 44) are obese.

Looking at the socio-demographic characteristics, we see that overweight and obese students are more often males, come more often from socio-economically disadvantaged families and are more likely to have a non-native background. Moreover, the share of students attending the Gymnasium is much lower among overweight (44%) and obese (36%) students compared to normal weight students (63%) (see S2 Table). This could be related to the fact that, as we will show below, obese students have lower academic performance than normal weight ones. In terms of psychological traits, overweight and obese students display a lower level of extraversion, conscientiousness, agreeableness, openness, and higher levels of neuroticism than normal weight students. However, given the relatively small share of obese students and the fact that some of these differences are pretty small, the only statistically significant differences are found on extraversion and conscientiousness. Instead, students with different BMI do not differ significantly in terms of attachment to school and time spent on homework.

**Fig 1:**
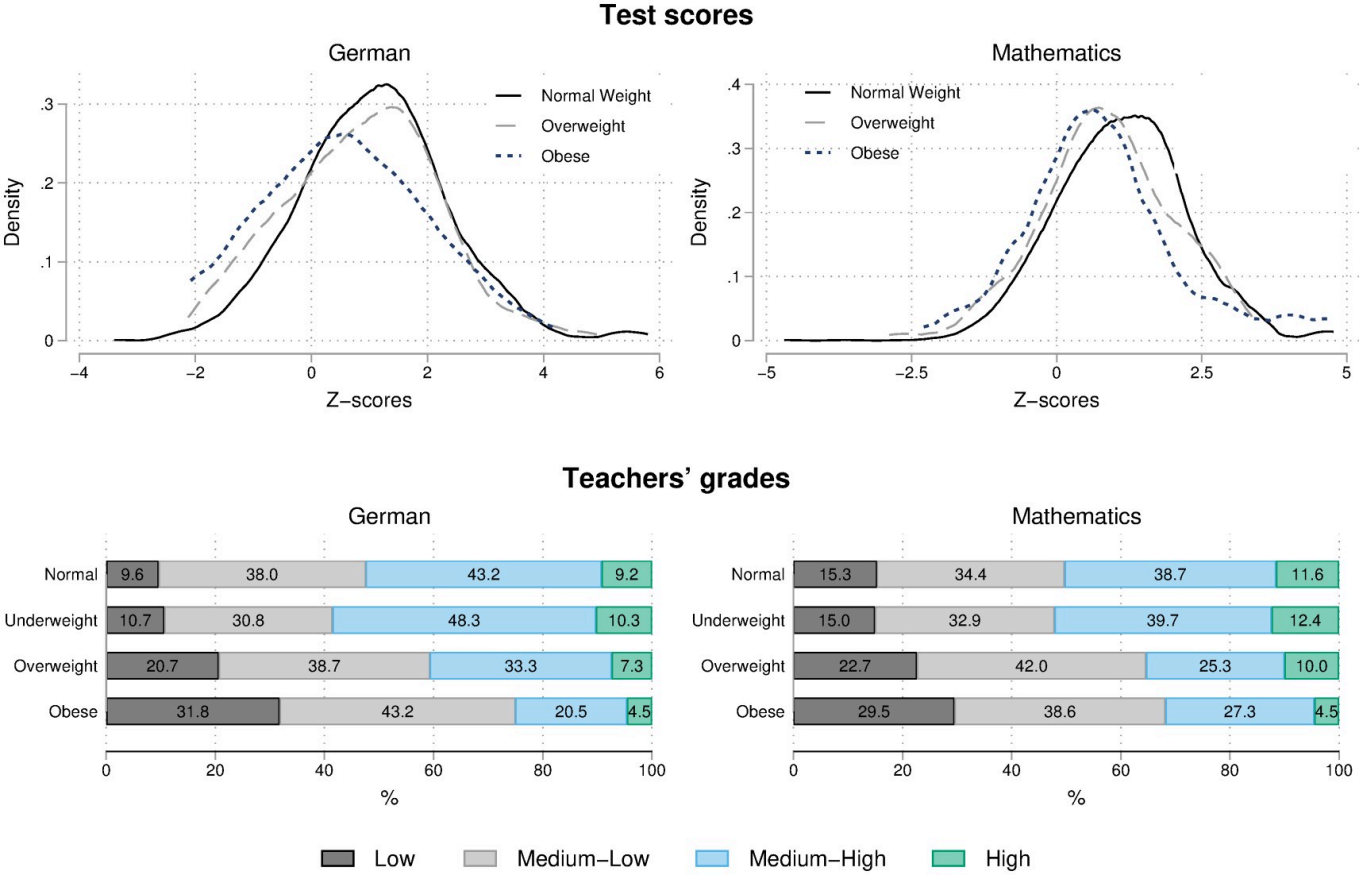
Distribution of test scores and teachers’ grades by students’ weight category. The upper-left graph shows Kernel density estimates of the distribution of test scores in German, the upper-right graph in mathematics. The lower-left graph shows the distribution of teachers’ grades by students’ weight category in German, and the lower-right graph in mathematics.

We now proceed by describing the bivariate relationship between students’ BMI and academic performance in lower secondary school in more detail. In the upper panel of Fig 1, the kernel density estimates of the distribution of students’ test scores in German (left) and mathematics (right) indicate that, on average, normal weight students have a higher performance than overweight ones, who in turn perform better than obese students. In the lower panel of Fig 1 we report the distribution of teachers’ grades in German (left) and mathematics (right) according the categories of BMI.

The patterns are similar across the two subjects: The incidence of children who received a low grade is largest among obese students, decreases but stays rather high among overweight students and is lower among normal weight students. The opposite pattern is found when looking at high grades, but in this case the difference between normal weight and obese students is more pronounced as when comparing normal weight to overweight students. Underweight students usually perform very similarly compared to normal weight students, either when academic performance is measured by teachers’ grades or standardized tests.

### Analysis of the grading bias against overweight and obese students

**Fig 2:**
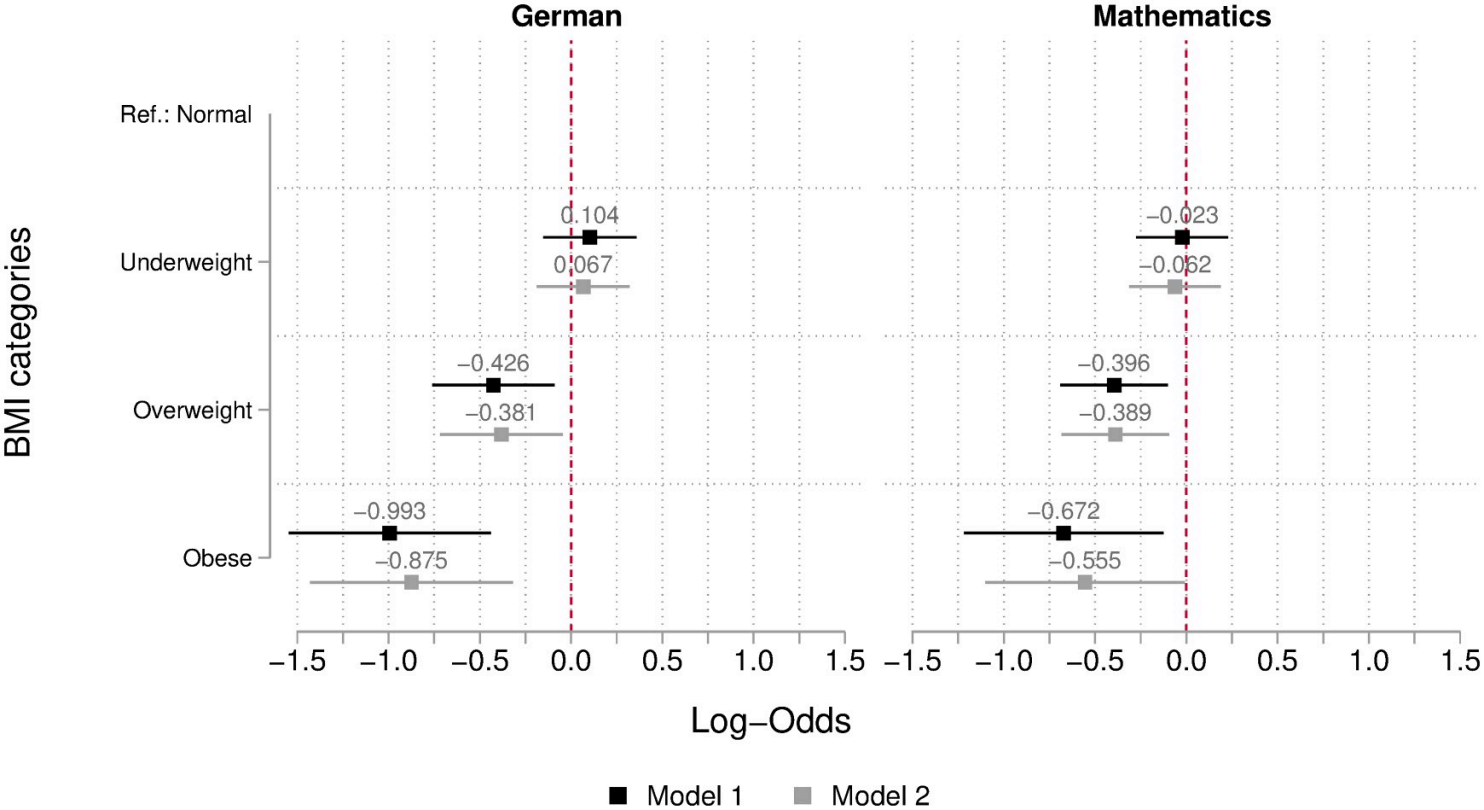
Teachers’ grading bias (measured in log-odds) according to students’ BMI. The graph shows log-odds ratios from the 3-level hierarchical ordered logit model on mathematics grades (left) and German (right). Bars represent 95% confidence intervals. Model 1 adjusts for students’ competence level and socio-demographic variables, while model 2 also includes students’ psychological traits, and school-related attitudes and behavior. N = 3,754. Analysis conducted on 50 multiply imputed datasets.

To assess whether overweight and obese students are less generously graded by their teachers compared to equally performing normal-weight students, we estimated a series of hierarchical ordered logistic regression models in which teachers’ grades are modelled as a function of students’ BMI categories, subject-specific performance in standardized tests, and a set of relevant individual characteristics. To correct for the missing values on relevant covariates, we conducted the analysis on 50 multiply imputed datasets. In Fig 2 we report the logit coefficients and 95% confidence intervals of the BMI categories of interest compared to normal weight students (the reference category). The complete models are reported in S3 Table. We report two model specifications: Model 1 adjusts for students’ competence level and socio-demographic variables, while Model 2 also includes students’ psychological traits, and school-related attitudes and behavior. However, since the two specifications lead to only minor differences in the estimated coefficients, in our comments we will focus on the results of the fully specified model.

The results indicate that underweight students are graded by their teachers very similarly to normal weight students: The estimates are close to zero and not statistically significant at the 95% confidence level. Conversely, overweight and obese students receive on average lower grades by their teachers than normal weight students with the same level of subject-specific competence. The disadvantages of obese students are larger than those of overweight students and are slightly more pronounced in German than in mathematics.

**Fig 3:**
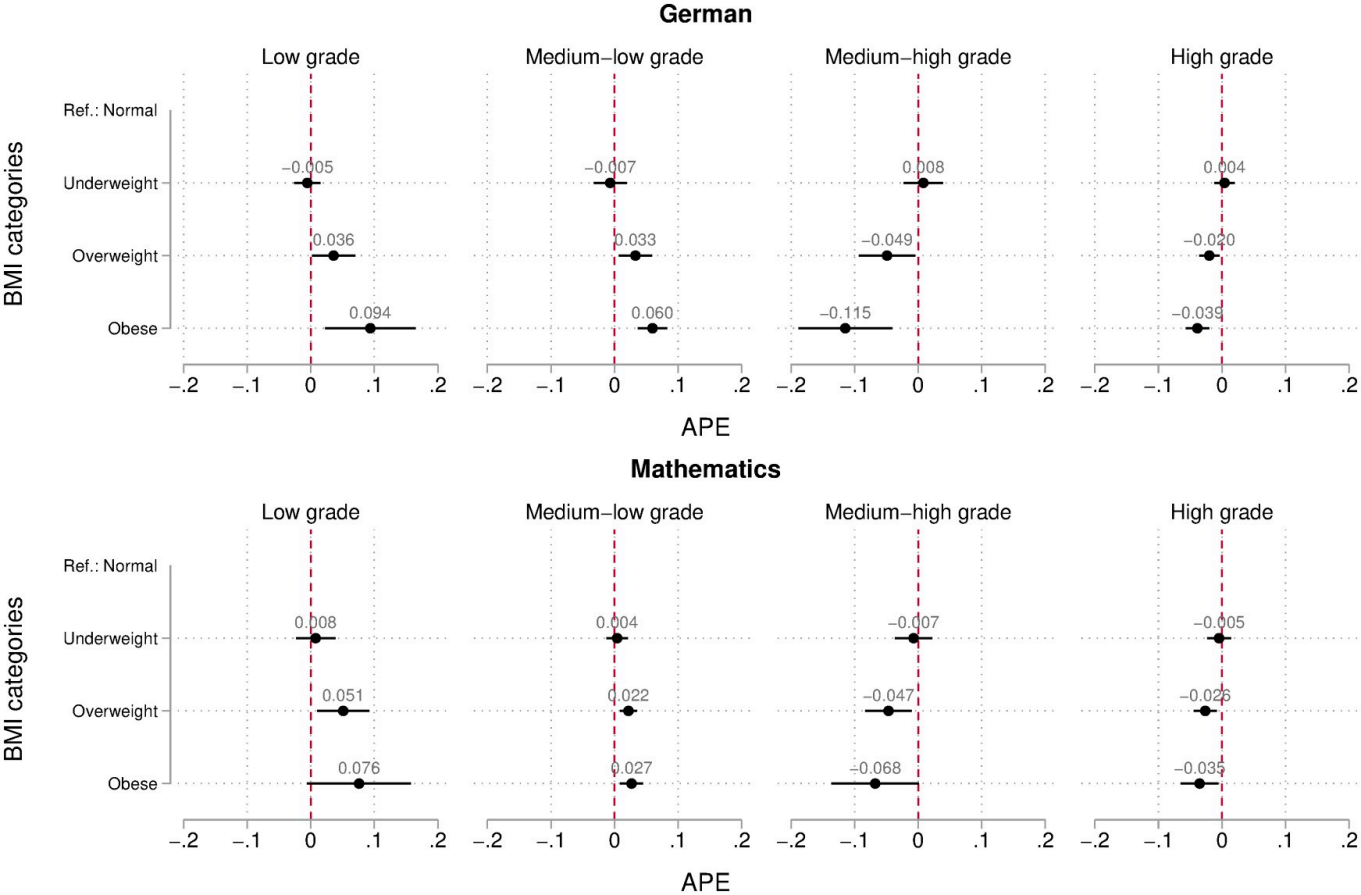
Teachers’ grading bias (measured in average partial effects) according to students’ BMI. The graph shows average partial effects from the 3-level hierarchical ordered logit model on mathematics grades (upper part) and German (lower part). Bars represent 95% confidence intervals. The models are adjusted for students’ competence level, socio-demographic characteristics, psychological traits, and school-related attitudes and behavior. N = 3,754. Analysis conducted on 50 multiply imputed datasets.

While relying on the log-odds gives us a synthetic picture of the overall relationship between BMI and teachers’ grading, it prevents us to quantify the strength of this relationship in a meaningful way. For this purpose, we present average partial effects of being underweight, overweight, or obese on the four levels of teachers’ grades in German (upper panel) and mathematics (lower panel) in Fig 3 (based on S5 Table). We see that, on average, overweight and obese students are more likely to receive low and medium-low grades, whereas they have lower chances to obtain high or medium-high grades compared to equally competent normal weight students with analogous socio-demographic characteristics, psychological traits, and school-related attitudes and behavior.

The largest differences among social groups are found at the lower end of the grade distribution and for medium-high grades. For instance, obese students have on average an 8 (mathematics) to 9 (German) percentage points larger probability of receiving a low grade by their teachers than equally competent normal weight students. They also have a lower probability of receiving medium-high (7–12 percentage points) and high (3–4 percentage points) grades than comparable normal weight students. Overweight is also associated to a penalization in grading, but its magnitude is smaller, ranging between 2 and 5 percentage points. From a qualitative point of view, the estimated differences appear to be larger in German than in mathematics, albeit the confidence intervals around the point estimates are to a large extent overlapped, making it difficult to state that the effect size across subjects actually differs in the reference population.

We estimated additional models in which we included an interaction term between the BMI categories and test scores, with the aim of assessing whether the grading bias differs among students with higher or lower levels of academic proficiency (see S7 Table). We found that this interaction is not statistically significant and estimated coefficients are substantially small, thus suggesting that the under-evaluation of overweight/obese students takes place at the same extent among low- and high-ability students.

### Heterogeneity in the overweight penalty by gender

As a last step, we investigate whether the penalty in grading associated with being overweight differs by gender, as found by a previous study in the US [9]. Given the small number of obese students, for this analysis we were forced to merge the categories of overweight and obese students together, in order to gain statistical power. Table 2 presents the results of the models that include an interaction between students’ BMI and gender (see S4 Table): More specifically, as suggested by Brambor et al. [77], we report the average partial effect of being overweight/obese versus normal weight on the probability of receiving different grades, among the group of females and males (adjusted for covariates included in the full models). In the last two rows we provide an estimate of the difference between these two effects, its corresponding standard error and information on whether it is statistically significant at different levels of confidence.

**Table 2:** Teachers’ grading bias (measured in average partial effects) over the grades distribution, according to students’ BMI and gender.

Effect of being overweight/ obese vs normal weight	German				Mathematics			
	Low	Medium-Low	Medium-High	High	Low	Medium-Low	Medium-High	High
Females	-0.006	-0.012	0.010	0.008	0.054	0.026	-0.051	-0.030
	(0.015)	(0.029)	(0.025)	(0.019)	(0.032)	(0.012)	(0.029)	(0.015)
Males	0.105***	0.064***	-0.129***	-0.040***	0.046	0.029*	-0.044*	-0.030*
	(0.025)	(0.009)	(0.026)	(0.007)	(0.023)	(0.012)	(0.022)	(0.013)
Δ_Male-Female_	0.110***	0.075*	-0.139***	-0.048*	-0.009	0.003	0.007	-0.000
	(0.029)	(0.030)	(0.035)	(0.020)	(0.039)	(0.016)	(0.036)	(0.019)

Note: The first two main rows present the average partial effect of being overweight/obese by gender and the last row reports a formal statistical test of the interaction BMI and gender. Standard errors in parentheses, statistical significance levels (* p<0.05; ** p<0.01; *** p<0.001). N = 2,194, underweight students are excluded from this analysis. 50 multiply imputed datasets.

**Fig 4:**
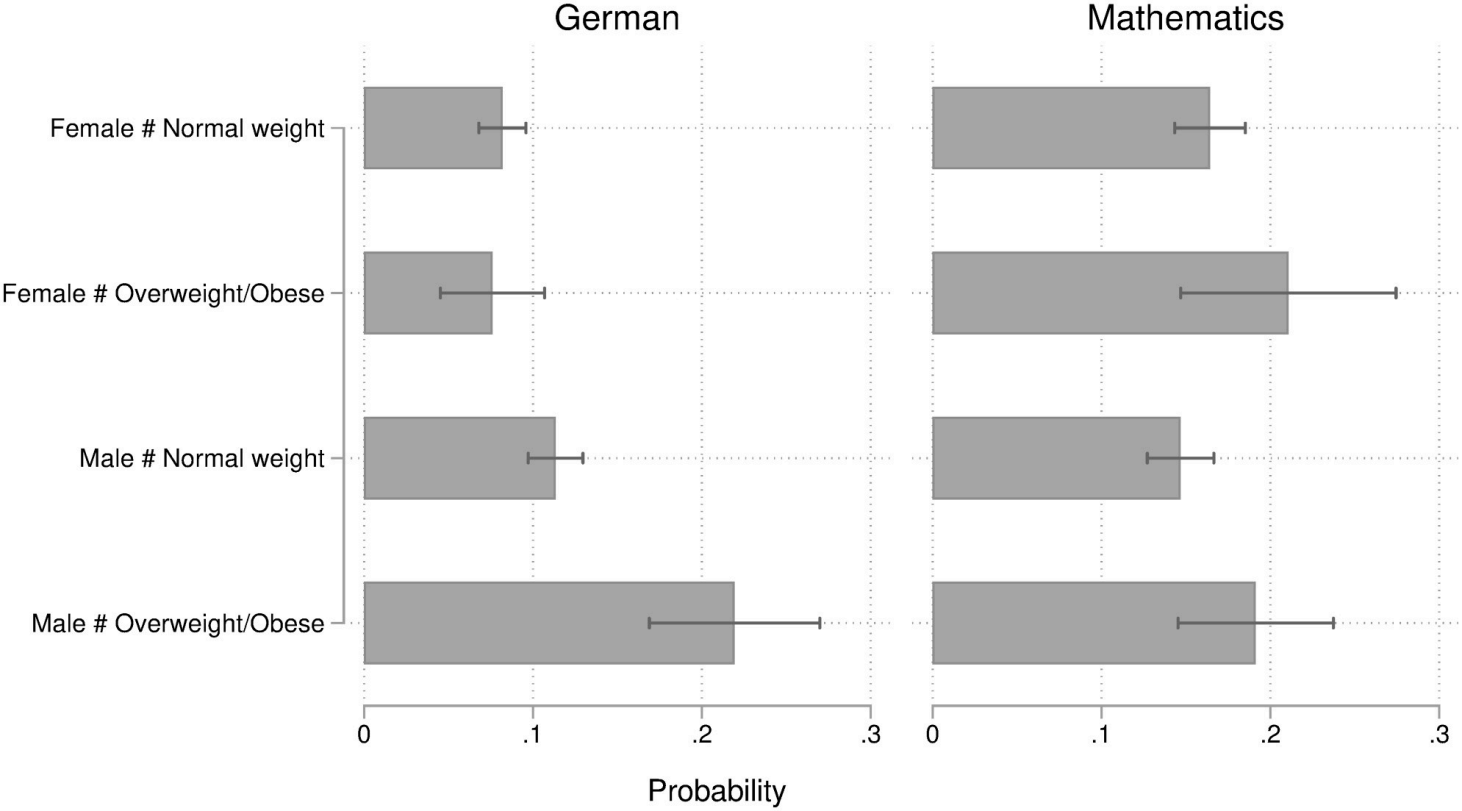
Predicted probabilities of obtaining a low grade by BMI and gender. Predicted probabilities (and 95% confidence intervals) derived from hierarchical ordinal logistic regression models of teachers’ grading in German (left graph) and mathematics (right graph), adjusted for students’ competence level, socio-demographic characteristics, psychological traits, and school-related attitudes and behavior. N = 3,754. Analysis conducted on 50 multiply imputed datasets.

Fig 4 (based on S6 Table) reports average predicted probabilities of obtaining a low grade by BMI and gender, derived from the same models. The results are pretty straightforward and in contrast to previous evidence from the US. Being overweight or obese is not related to any penalization in grading among female students. Consequently, the penalty observed in the main analysis is mostly due to teachers’ grading of male students. The pattern is particularly pronounced in German: Among males, indeed, overweight/obese students have a much higher probability of receiving a low grade (+11 percentage points) compared to normal weight students with the same subject-specific proficiency, psychological traits, and school-related attitudes and behavior. Overweight/obese males also have a substantially lower probability of getting medium-high (-13 percentage points) and high (-4 percentage points) grades in German.

The penalization in German due to being overweight/obese among males is substantially larger than among females, ranging from 5 to 14 percentage points, and statistically significant across all the four grades. In mathematics, the qualitative pattern differs to some extent: Albeit we detected statistically significant effects of being overweight only among males (ranging between 2 and 4 percentage points), the effect size among females is very similar. The differences in weight penalty by gender are not statistically significant in mathematics. Thus, given the empirical evidence, we cannot reject the null hypothesis of no difference in the grading penalization against overweight/obese students between boys and girls in the reference population.

## Discussion and conclusions

From previous studies we knew that overweight students get, on average, lower grades than their peers in school [30, 31] but it was not clear if the differences in grades stem from discrimination or from actual differences in competences. One should expect that students with the same demonstrated competence in a given subject, and same school-related attitudes and behavior will get similar grades from their teachers if fair grading is in place. We asked if lower mathematics and German grades are attributed to overweight compared to normal weight students, even when they have the same level of subject-specific competence, main psychological traits, and school-related attitudes and behavior. The focus on students in German lower secondary education allows us to concentrate on a key developmental stage within an underexplored country context, in which obesity could lead to stronger stigma due to the comparatively lower incidence of this phenomenon.

By means of hierarchical ordinal logistic regression models we have shown that both, overweight and obese students, receive on average lower grades than comparatively similar normal weight students, thus corroborating our first hypothesis. This pattern is found irrespective of whether we adjust for basic socio-demographic characteristics alone or for a more extensive set of individuals’ features including students’ personality traits, school-related attitudes and behavior.

While it is difficult to strictly prove “discrimination” within an observational setting, the rich set of control variables and the rather large size of the estimated differences seem to suggest that overweight, and especially obese students, are subject to some forms of penalty in the evaluations they receive by their teachers. The penalization is not only statistically significant, but also substantial. Indeed, the effect size of being obese rather than normal weight is larger than that of being male against being female, a comparison that has attracted much more attention among scholars [e.g., 78]. It is important to note two additional aspects. First, the consequences of being overweight and obese cannot be ascribed to a simple deviance-from-the-norm effect since underweight students do not experience any penalty in teachers’ evaluations. Second, considering a wide array of students’ traits, attitudes and behavior does not alter substantially the estimating teacher grading bias, pointing to the possibility that a large part of the total teacher grading bias stems from discrimination and stigmatization processes.

Empirical evidence is instead less clear in supporting our second hypothesis, which states that the grading penalty should be larger in German than in mathematics. In the common wisdom, math teachers are expected to base decisions on grades on a certain score that is linked to pre-determined correct solutions. Consequently, one might argue that math exams leave less room for interpretation regarding which grades the students should receive. Apart from that, teachers may set a variety of exams in the subject German with a different scope for interpretation if a result is right or wrong. While dictation exercises are solved either correctly or incorrectly, there is not a unique correct answer in essays and text comprehension exercises, and therefore the grade could incorporate subjective sources of bias. We found that, from a qualitative point of view, the effect sizes associated to being obese or overweight across subjects follows the expected pattern. From a statistical inference point of view, the confidence intervals around the point estimates are instead rather large and overlapped to a large extent, thus not supporting the hypothesis of differential effects across subjects. Anyway, our findings suggest that–despite mathematics being usually considered a more objective subject–this discipline is also not exempted from teachers’ grading bias linked to body appearance.

Additional insights on the heterogeneity in the penalization of overweight and obese students come from the second analysis, in which we investigate whether gender moderates the effect of BMI. Following theoretical arguments on the role of femininity in contemporary Western societies and previous findings from the US, we expected a larger detrimental effect of obesity on grades among females. Contrarily, we found that the more severe grading to which overweight/obese students are exposed in lower secondary school is mainly concentrated among male students, a pattern that is particularly pronounced in the German subject and less in mathematics. Differently from what was observed in the US, in Germany it seems that body appearance linked to negative stereotypes amplifies the stricter grading standards to which males are already exposed in comparison to females. While this might be due to processes of statistical discrimination by teachers, part of this result might be linked to individuals’ behavior in classrooms that we are not able to capture with our variables. If the two stereotypes of boys being less diligent than girls [79] and overweight people being less disciplined than normal weight people are simultaneously present, this can lead to a double disadvantage for overweight boys. For overweight girls, in contrast, the gender stereotype of being more diligent can counteract the weight stereotype of being less disciplined, leading to lower bias. The latter might be especially relevant in a subject like German, where femininity is a favorable attribute.

Before concluding with policy recommendations, it is important to discuss some limitations. First, the grades are self-reported which means that the outcome variable can suffer from measurement error if the students intentionally or unintentionally reported wrong grades. Yet, we do not believe this issue should be a major concern, for two reasons. First, although especially students with unfavorable grades might have social desirability biases and report a better grade to increase their self-esteem, self-reported grades were found to be very similar to grades in the report cards in previous studies [80]. Second, if overweight/obese students on average obtain lower grades and are thus, on average, more prone to increase their self-reported grades, this could lead to an underestimation of their penalty. If this is the case, our findings would be based on conservative estimates.

The second limitation lies within the cross-sectional nature of the study, which does not allow us to disentangle whether overweight causes lower grades or if lower grades cause overweight by leading to stress eating [51]. Nevertheless, we believe that even if there are self-reciprocating effects, on theoretical ground it is much more plausible that the largest effect is of BMI on grades and not *vice versa*. Furthermore, additional models in which we adjusted for life satisfaction to rule out extreme stress provide similar results.

The last limitation refers to the main assumption behind the grade-equation models, which posits that standardized test scores act as a good proxy for latent subject-specific competences. This assumption could be questioned if some unobserved factors associated with student BMI also affect the idiosyncratic performance in the standardized test. We are not aware of specific studies that try to tackle this aspect in-depth. However, results from previous studies on teachers’ grading bias against immigrants or boys using a grade-equation model [76] report qualitatively similar findings than the few studies employing experimental designs [81].

Despite these limitations, we believe our study contributed to the literature by showing that overweight students have lower grades in German which can neither be attributed to lower subject-specific competence nor to psychological characteristics or key school-related attitudes and behavior. If institutions use grades as information on the competence of an applicant [82, 83], they should not contain anything else than academic competence in the given subject to be a valid source [10, 83]. But in reality, grades include other more subjective measures besides competences [10], and this can penalize students with the same competences but lower grades. To decrease the influence of discrimination and physical appearance on grades, we point towards a variety of educational policies at different levels of intervention. At the macro-level, our findings suggest the possibility of using competence test scores together with teachers’ grades to provide an overall assessment of students’ academic proficiency, especially at the end of educational cycles or in proximity of important educational transitions. At the meso- and micro-level, our findings point towards possible interventions on the grading policies and practices adopted by schools and teachers. First, in line with other scholars’ advise [34], it appears to be important to grade students not in comparison to their classmates but to external criteria. These criteria should be stated in class and include what the students are expected to achieve and how the proof of achievement looks like [11]. Additionally, aside from competence-oriented practices, the censoring of the students’ names on exams could decrease bias due to personal characteristics [81]. Another possible suggestion are training courses for raising the teachers’ awareness of unconscious bias. This could enable them to detect and counteract discriminatory behavior. Although, awareness training in the context of grading is not researched by now, there are hints that raising awareness might lower bias in general: Indeed, it was found that people who believe to be probably biased against women actually judge them in a less biased way [84]. Not only for overweight students, but also for other discriminated minorities, external and fixed standards as well as increased awareness of possible discrimination might offer the opportunity to reduce inequalities of opportunities in the educational context.

## Supporting information

S1 Table Distribution of missing values across the variables (N = 3,814). (DOCX)

S2 Table Distribution of attributes in the analytical sample of students (N = 2,142). (DOCX)

S3 Table Three-level hierarchical ordinal logistic regression model: Log-odds ratio on teachers’ grades. (DOCX)

S4 Table Three-level hierarchical ordinal logistic regression model with interaction: Log-odds ratio on teachers’ grades. (DOCX)

S5 Table Average partial effect (APE) estimates reported in Fig 3. (DOCX)

S6 Table Average partial effect (APE) estimates reported in Fig 4. (DOCX)

S7 Table Interaction between BMI categories and students’ test scores (N = 3,814). (DOCX)
