# Supplementary material for: The weight of school grades: Evidence of biased teachers’ evaluations against overweight students in Germany
Source: PLoS One. 2021 Feb 8;16(2):e0245972. doi: 10.1371/journal.pone.0245972 (PMC7869982; doi:10.1371/journal.pone.0245972)
Supplement: S1 Table — (DOCX) [file pone.0245972.s001.docx]

**S1 Table. Distribution of missing values across the variables (N=3,814).**

| **Variable** | **Frequency missing** | **% missing** |
| --- | --- | --- |
| BMI | 959 | 25.1 |
| German grade | 282 | 7.4 |
| Mathematics grade | 269 | 7.1 |
| Attachment to school | 201 | 5.3 |
| Homework duration | 166 | 4.4 |
| Extraversion | 134 | 3.5 |
| Agreeableness | 122 | 3.2 |
| Parental ISEI | 116 | 3.0 |
| Neuroticism | 90 | 2.4 |
| Openness | 70 | 1.8 |
| Conscientiousness | 68 | 1.8 |
| Parental ISCED | 67 | 1.8 |
| Reading competence (test scores) | 4 | 0.1 |
| Mathematics competence (test scores) | 1 | 0.0 |
| Gender | 0 | 0.0 |
| Age in years | 0 | 0.0 |
| Native language | 0 | 0.0 |
| School type | 0 | 0.0 |
| School region | 0 | 0.0 |
