# Supplementary material for: The weight of school grades: Evidence of biased teachers’ evaluations against overweight students in Germany
Source: PLoS One. 2021 Feb 8;16(2):e0245972. doi: 10.1371/journal.pone.0245972 (PMC7869982; doi:10.1371/journal.pone.0245972)
Supplement: S2 Table — (DOCX) [file pone.0245972.s002.docx]

**S2 Table. Distribution of attributes in the analytical sample of students (N=2,142).**

|  |  | **% per BMI category** | | | | **% total** |
| --- | --- | --- | --- | --- | --- | --- |
|  |  |  | **Under-** | **Over-** |  | **All** |
| **Attributes** | **Categories** | **Healthy** | **weight** | **weight** | **Obese** | **students** |
| German grade | Low | 9.4 | 10.4 | 20.1 | 31.8 | 10.7 |
|  | Medium-low | 38.1 | 30.4 | 38.9 | 43.2 | 37.4 |
|  | Medium-high | 43.2 | 48.7 | 33.6 | 20.5 | 42.7 |
|  | High | 9.3 | 10.4 | 7.4 | 4.5 | 9.2 |
| Mathematics grade | Low | 15.1 | 15.2 | 22.1 | 29.5 | 15.9 |
|  | Medium-low | 34.5 | 32.2 | 42.3 | 38.6 | 34.9 |
|  | Medium-high | 38.7 | 40.0 | 25.5 | 27.3 | 37.7 |
|  | High | 11.7 | 12.6 | 10.1 | 4.5 | 11.5 |
| BMI categories | Normal weight | 100.0 | 0.0 | 0.0 | 0.0 | 80.3 |
|  | Underweight | 0.0 | 100.0 | 0.0 | 0.0 | 10.7 |
|  | Overweight | 0.0 | 0.0 | 100.0 | 0.0 | 7.0 |
|  | Obese | 0.0 | 0.0 | 0.0 | 100.0 | 2.1 |
| Reading competence (test scores) |  | 1.101 | 0.889 | 0.877 | 0.564 | 1.052 |
| Mathematics competence (test scores) |  | 1.133 | 1.087 | 0.867 | 0.814 | 1.103 |
| Gender | Female | 50.4 | 48.7 | 36.2 | 36.4 | 49.0 |
|  | Male | 49.6 | 51.3 | 63.8 | 63.6 | 51.0 |
| Age in years | 12 | 55.7 | 61.3 | 53.7 | 56.8 | 56.2 |
|  | 13 | 44.3 | 38.7 | 46.3 | 43.2 | 43.8 |
| Parental ISCED | 2 or lower | 3.2 | 1.7 | 9.4 | 6.8 | 3.5 |
|  | 3b | 25.4 | 23.5 | 34.9 | 36.4 | 26.1 |
|  | 3a & 3c | 3.3 | 2.2 | 4.0 | 0.0 | 3.1 |
|  | 4a & 5b | 31.1 | 26.1 | 28.2 | 29.5 | 30.3 |
|  | 5a & 6 | 37.0 | 46.5 | 23.5 | 27.3 | 36.9 |
| Parental ISEI |  | 55.5 | 58.3 | 51.1 | 49.9 | 55.4 |
| Native language | German only | 79.7 | 82.6 | 77.9 | 72.7 | 79.7 |
|  | Other native language | 20.3 | 17.4 | 22.1 | 27.3 | 20.3 |
| School type | Hauptschule | 4.2 | 5.2 | 6.0 | 13.6 | 4.6 |
|  | Realschule | 19.7 | 19.1 | 27.5 | 20.5 | 20.2 |
|  | Gymnasium | 62.8 | 62.2 | 43.6 | 36.4 | 60.9 |
|  | School with different tracks | 13.3 | 13.5 | 22.8 | 29.5 | 14.3 |
| School region | West | 83.8 | 84.8 | 76.5 | 81.8 | 83.4 |
|  | East | 16.2 | 15.2 | 23.5 | 18.2 | 16.6 |
| Age |  | 12.4 | 12.4 | 12.5 | 12.4 | 12.4 |
| Extraversion (std.) |  | 0.076 | 0.100 | -0.149 | -0.159 | 0.058 |
| Agreeableness (std.) |  | -0.011 | 0.110 | -0.071 | -0.105 | -0.004 |
| Conscientiousness (std.) |  | 0.024 | 0.139 | -0.15 | -0.259 | 0.019 |
| Neuroticism (std.) |  | -0.031 | -0.094 | -0.083 | 0.399 | -0.033 |
| Openness (std.) |  | 0.028 | 0.049 | -0.109 | -0.123 | 0.017 |
| Attachment to school (std.) |  | 0.042 | 0.038 | -0.061 | -0.109 | 0.031 |
| Homework duration (std.) |  | 0.002 | 0.004 | -0.005 | -0.210 | -0.003 |

Descriptive statistics are computed on the analytical sample without the imputation.
