# Supplementary material for: The weight of school grades: Evidence of biased teachers’ evaluations against overweight students in Germany
Source: PLoS One. 2021 Feb 8;16(2):e0245972. doi: 10.1371/journal.pone.0245972 (PMC7869982; doi:10.1371/journal.pone.0245972)
Supplement: S3 Table — (DOCX) [file pone.0245972.s003.docx]

**S3 Table. Three-level hierarchical ordinal logistic regression model: log-odds ratio on teachers' grades.**

|  | **German** | | **Mathematics** | |
| --- | --- | --- | --- | --- |
| **Teachers' grades** | **Model 1** | **Model 2** | **Model 3** | **Model 4** |
| *BMI categories (ref.: normal weight)* |  |  |  |  |
| Underweight | 0.104 | 0.067 | -0.023 | -0.062 |
|  | (0.130) | (0.129) | (0.128) | (0.128) |
| Overweight | -0.426* | -0.381* | -0.396** | -0.389** |
|  | (0.170) | (0.171) | (0.150) | (0.150) |
| Obese | -0.993*** | -0.875** | -0.672* | -0.555* |
|  | (0.281) | (0.282) | (0.277) | (0.278) |
| Male (ref.: female) | -0.707*** | -0.599*** | 0.162* | 0.218** |
|  | (0.073) | (0.079) | (0.070) | (0.075) |
| *Age in years (ref.: ≤ 10)* |  |  |  |  |
| 11 | -2.576* | -2.349 | 0.356 | 0.473 |
|  | (1.221) | (1.213) | (1.115) | (1.102) |
| 12 | -2.726* | -2.539* | 0.168 | 0.230 |
|  | (1.184) | (1.176) | (1.078) | (1.065) |
| 13 | -2.814* | -2.608* | 0.077 | 0.141 |
|  | (1.185) | (1.176) | (1.078) | (1.065) |
| 14 | -3.018* | -2.871* | -0.041 | -0.043 |
|  | (1.190) | (1.182) | (1.085) | (1.072) |
| ≥ 15 | -3.418** | -3.309** | 0.505 | 0.433 |
|  | (1.284) | (1.276) | (1.190) | (1.185) |
| Parental SES | 0.002 | 0.001 | 0.000 | 0.000 |
|  | (0.003) | (0.003) | (0.003) | (0.003) |
| *Parental ISCED (ref.: 2 or lower)* |  |  |  |  |
| 3b | 0.023 | 0.053 | -0.023 | -0.018 |
|  | (0.189) | (0.190) | (0.183) | (0.185) |
| 3a & 3c | 0.342 | 0.310 | 0.174 | 0.108 |
|  | (0.253) | (0.254) | (0.247) | (0.250) |
| 4a & 5b | 0.357 | 0.382 | 0.109 | 0.110 |
|  | (0.196) | (0.198) | (0.187) | (0.189) |
| 5a & 6 | 0.640** | 0.668** | 0.340 | 0.351 |
|  | (0.214) | (0.215) | (0.203) | (0.205) |
| Other native language (ref.: German only) | -0.068 | -0.073 | -0.065 | -0.05 |
|  | (0.091) | (0.092) | (0.089) | (0.089) |
| *School type (ref.: Hauptschule)* |  |  |  |  |
| Realschule | -0.201 | -0.166 | -0.593** | -0.525** |
|  | (0.189) | (0.192) | (0.193) | (0.194) |
| Gymnasium | 0.223 | 0.224 | -0.699*** | -0.674*** |
|  | (0.187) | (0.190) | (0.191) | (0.193) |
| School with different tracks | 0.017 | 0.009 | -0.460* | -0.416 |
|  | (0.211) | (0.213) | (0.212) | (0.213) |
| East (ref.: west) | 0.952*** | 1.015*** | 0.802*** | 0.846*** |
|  | (0.156) | (0.158) | (0.155) | (0.157) |
| Extraversion (std.) |  | 0.144*** |  | -0.022 |
|  |  | (0.037) |  | (0.037) |
| Agreeableness (std.) |  | 0.017 |  | -0.018 |
|  |  | (0.039) |  | (0.037) |
| Conscientiousness (std.) |  | 0.398*** |  | 0.354*** |
|  |  | (0.039) |  | (0.038) |
| Neuroticism (std.) |  | -0.040 |  | -0.072* |
|  |  | (0.038) |  | (0.036) |
| Openness (std.) |  | 0.032 |  | -0.107** |
|  |  | (0.037) |  | (0.035) |
| Attachment to school (std.) |  | 0.074* |  | 0.091* |
|  |  | (0.037) |  | (0.036) |
| Homework duration (std.) |  | -0.118** |  | -0.073* |
|  |  | (0.037) |  | (0.036) |
| Reading competence (test scores) | 0.507*** | 0.506*** |  |  |
|  | (0.032) | (0.033) |  |  |
| Mathematics competence (test scores) |  |  | 0.784*** | 0.798*** |
|  |  |  | (0.038) | (0.039) |
| Cut 1 | -4.651*** | -4.483*** | -1.303 | -1.233 |
|  | (1.210) | (1.202) | (1.102) | (1.090) |
| Cut 2 | -1.969 | -1.716 | 0.752 | 0.878 |
|  | (1.208) | (1.200) | (1.101) | (1.089) |
| Cut 3 | 1.049 | 1.396 | 3.288** | 3.476** |
|  | (1.207) | (1.200) | (1.102) | (1.090) |
| *Variance components* |  |  |  |  |
| Individual | 0.219** | 0.239*** | 0.307*** | 0.310*** |
|  | (0.068) | (0.067) | (0.064) | (0.064) |
| German class | 0.208** | 0.178* |  |  |
|  | (0.076) | (0.073) |  |  |
| Mathematics class |  |  | 0.085 | 0.085 |
|  |  |  | (0.056) | (0.056) |
| *N* | 3,754 | 3,754 | 3,754 | 3,754 |

Standard errors in parentheses. * p < 0.05, ** p < 0.01, *** p < 0.001
