# Supplementary material for: The weight of school grades: Evidence of biased teachers’ evaluations against overweight students in Germany
Source: PLoS One. 2021 Feb 8;16(2):e0245972. doi: 10.1371/journal.pone.0245972 (PMC7869982; doi:10.1371/journal.pone.0245972)
Supplement: S4 Table — (DOCX) [file pone.0245972.s004.docx]

**S4 Table.** **Three-level hierarchical ordinal logistic regression model with interaction: log-odds ratio on teachers' grades.**

|  | **German** | | **Mathematics** | |
| --- | --- | --- | --- | --- |
| **Teachers' grades** | **Model 1** | **Model 2** | **Model 3** | **Model 4** |
| Overweight/obese (ref.: normal weight) | 0.019 | 0.099 | -0.496* | -0.432 |
|  | (0.231) | (0.242) | (0.223) | (0.237) |
| Male (ref.: female) | -0.539*** | -0.414*** | 0.108 | 0.164 |
|  | (0.091) | (0.101) | (0.087) | (0.099) |
| Overweight/obese × male | -0.959** | -1.067*** | 0.097 | 0.039 |
|  | (0.293) | (0.308) | (0.280) | (0.296) |
| *Age in years (ref.: ≤ 10)* |  |  |  |  |
| 11 | -3.119 | -3.028 | -0.298 | -0.036 |
|  | (1.635) | (1.638) | (1.334) | (1.354) |
| 12 | -3.246* | -3.186* | -0.611 | -0.511 |
|  | (1.596) | (1.595) | (1.288) | (1.304) |
| 13 | -3.392* | -3.320* | -0.756 | -0.688 |
|  | (1.596) | (1.595) | (1.288) | (1.305) |
| 14 | -3.449* | -3.487* | -0.653 | -0.717 |
|  | (1.603) | (1.603) | (1.297) | (1.314) |
| ≥ 15 | -3.706* | -3.469* | -0.237 | -0.574 |
|  | (1.697) | (1.704) | (1.405) | (1.431) |
| Parental SES | 0.003 | 0.003 | 0.002 | 0.002 |
|  | (0.003) | (0.004) | (0.003) | (0.003) |
| *Parental ISCED (ref.: 2 or lower)* |  |  |  |  |
| 3b | -0.043 | -0.106 | -0.192 | -0.226 |
|  | (0.238) | (0.251) | (0.226) | (0.241) |
| 3a & 3c | 0.379 | 0.403 | -0.212 | -0.288 |
|  | (0.313) | (0.324) | (0.298) | (0.317) |
| 4a & 5b | 0.335 | 0.303 | -0.087 | -0.097 |
|  | (0.245) | (0.256) | (0.230) | (0.244) |
| 5a & 6 | 0.620* | 0.614* | 0.16 | 0.119 |
|  | (0.264) | (0.277) | (0.248) | (0.263) |
| Other native language (ref.: German only) | -0.095 | -0.155 | -0.124 | -0.136 |
|  | (0.112) | (0.118) | (0.108) | (0.115) |
| *School type (ref.: Hauptschule)* |  |  |  |  |
| Realschule | 0.050 | 0.087 | -0.357 | -0.240 |
|  | (0.234) | (0.255) | (0.236) | (0.259) |
| Gymnasium | 0.443 | 0.474 | -0.445 | -0.358 |
|  | (0.234) | (0.255) | (0.237) | (0.261) |
| School with different tracks | 0.262 | 0.275 | -0.172 | -0.071 |
|  | (0.260) | (0.282) | (0.260) | (0.284) |
| East (ref.: west) | 1.136*** | 1.185*** | 0.837*** | 0.911*** |
|  | (0.170) | (0.176) | (0.170) | (0.180) |
| Extraversion |  | 0.131* |  | 0.002 |
|  |  | (0.059) |  | (0.057) |
| Agreeableness |  | -0.074 |  | -0.081 |
|  |  | (0.076) |  | (0.072) |
| Conscientiousness |  | 0.521*** |  | 0.424*** |
|  |  | (0.060) |  | (0.057) |
| Neuroticism |  | -0.029 |  | -0.084 |
|  |  | (0.057) |  | (0.055) |
| Openness |  | 0.033 |  | -0.137** |
|  |  | (0.048) |  | (0.047) |
| Attachment to school |  | 0.047 |  | 0.060 |
|  |  | (0.043) |  | (0.041) |
| Homework duration |  | -0.171*** |  | -0.046 |
|  |  | (0.048) |  | (0.046) |
| Reading competence (test scores) | 0.527*** | 0.519*** |  |  |
|  | (0.039) | (0.042) |  |  |
| Mathematics competence (test scores) |  |  | 0.784*** | 0.822*** |
|  |  |  | (0.047) | (0.051) |
| Cut 1 | -4.873** | -3.167 | -2.060 | -1.384 |
|  | (1.620) | (1.672) | (1.316) | (1.396) |
| Cut 2 | -2.116 | -0.340 | 0.047 | 0.804 |
|  | (1.619) | (1.671) | (1.314) | (1.394) |
| Cut 3 | 0.885 | 2.753 | 2.532 | 3.392* |
|  | (1.618) | (1.671) | (1.315) | (1.395) |
| *Variance components* |  |  |  |  |
| Individual | 0.161 | 0.183* | 0.309*** | 0.332*** |
|  | (0.085) | (0.088) | (0.078) | (0.089) |
| German class | 0.243* | 0.212 |  |  |
|  | (0.106) | (0.109) |  |  |
| Mathematics class |  |  | 0.039 | 0.071 |
|  |  |  | (0.071) | (0.083) |
| *N* | 2,500 | 2,238 | 2,500 | 2,238 |

Standard errors in parentheses. * p < 0.05, ** p < 0.01, *** p < 0.001
