# Supplementary material for: The weight of school grades: Evidence of biased teachers’ evaluations against overweight students in Germany
Source: PLoS One. 2021 Feb 8;16(2):e0245972. doi: 10.1371/journal.pone.0245972 (PMC7869982; doi:10.1371/journal.pone.0245972)
Supplement: S5 Table — (DOCX) [file pone.0245972.s005.docx]

**S5 Table. Average partial effect (APE) estimates reported in Fig 3.**

|  | **German** | | | **Mathematics** | | |
| --- | --- | --- | --- | --- | --- | --- |
|  | **Estimate** | **SE** | **p-value** | **Estimate** | **SE** | **p-value** |
| *Teachers’ grade: Low* |  |  |  |  |  |  |
| Underweight | -0.005 | 0.011 | 0.608 | 0.008 | 0.016 | 0.623 |
| Overweight | 0.036 | 0.017 | 0.039 | 0.051 | 0.021 | 0.015 |
| Obese | 0.094 | 0.036 | 0.010 | 0.076 | 0.042 | 0.068 |
| *Teachers’ grade: Medium-low* |  |  |  |  |  |  |
| Underweight | -0.007 | 0.013 | 0.607 | 0.004 | 0.009 | 0.630 |
| Overweight | 0.033 | 0.013 | 0.014 | 0.022 | 0.007 | 0.002 |
| Obese | 0.060 | 0.012 | 0.000 | 0.027 | 0.009 | 0.004 |
| *Teachers’ grade: Medium-high* |  |  |  |  |  |  |
| Underweight | 0.008 | 0.016 | 0.608 | -0.007 | 0.015 | 0.624 |
| Overweight | -0.049 | 0.023 | 0.030 | -0.047 | 0.019 | 0.012 |
| Obese | -0.115 | 0.038 | 0.002 | -0.068 | 0.035 | 0.053 |
| *Teachers’ grade: High* |  |  |  |  |  |  |
| Underweight | 0.004 | 0.008 | 0.607 | -0.005 | 0.009 | 0.628 |
| Overweight | -0.020 | 0.008 | 0.014 | -0.026 | 0.009 | 0.004 |
| Obese | -0.039 | 0.009 | 0.000 | -0.035 | 0.015 | 0.020 |
