# Supplementary material for: The weight of school grades: Evidence of biased teachers’ evaluations against overweight students in Germany
Source: PLoS One. 2021 Feb 8;16(2):e0245972. doi: 10.1371/journal.pone.0245972 (PMC7869982; doi:10.1371/journal.pone.0245972)
Supplement: S6 Table — (DOCX) [file pone.0245972.s006.docx]

**S6 Table. Average partial effect (APE) estimates reported in Fig 4.**

|  |  |  | |  | |  | | **95% confidence interval** | |
| --- | --- | --- | --- | --- | --- | --- | --- | --- | --- |
|  | **Margin** | | **SE** | | **t** | | **p-value** | **Low bound** | **High bound** |
| **German** |  | |  | |  | |  |  |  |
| Female × normal weight | 0.082 | | 0.007 | | 11.530 | | 0.000 | 0.068 | 0.096 |
| Female × overweight/obese | 0.076 | | 0.016 | | 4.840 | | 0.000 | 0.045 | 0.107 |
| Male × normal weight | 0.113 | | 0.008 | | 13.700 | | 0.000 | 0.097 | 0.130 |
| Male × overweight/obese | 0.219 | | 0.026 | | 8.520 | | 0.000 | 0.169 | 0.270 |
| **Mathematics** |  | |  | |  | |  |  |  |
| Female × normal weight | 0.164 | | 0.011 | | 15.400 | | 0.000 | 0.143 | 0.185 |
| Female × overweight/obese | 0.211 | | 0.033 | | 6.480 | | 0.000 | 0.147 | 0.274 |
| Male × normal weight | 0.147 | | 0.010 | | 14.620 | | 0.000 | 0.127 | 0.167 |
| Male × overweight/obese | 0.191 | | 0.023 | | 8.160 | | 0.000 | 0.145 | 0.237 |
