# Supplementary material for: The weight of school grades: Evidence of biased teachers’ evaluations against overweight students in Germany
Source: PLoS One. 2021 Feb 8;16(2):e0245972. doi: 10.1371/journal.pone.0245972 (PMC7869982; doi:10.1371/journal.pone.0245972)
Supplement: S7 Table — (DOCX) [file pone.0245972.s007.docx]

**S7 Table. Interaction between BMI categories and students’ test scores (N=3,814).**

|  | **Estimate** | **SD** | **t-value** | **p-value** | **95%CI: min** | **95%CI: max** |
| --- | --- | --- | --- | --- | --- | --- |
| **German** |  |  |  |  |  |  |
| Underweight | 0.040 | 0.149 | 0.270 | 0.788 | -0.253 | 0.334 |
| Overweight | -0.406 | 0.200 | -2.030 | 0.044 | -0.800 | -0.011 |
| Obese | -0.860 | 0.304 | -2.830 | 0.005 | -1.459 | -0.261 |
|  |  |  |  |  |  |  |
| German test scores | 0.517 | 0.035 | 14.630 | 0.000 | 0.447 | 0.586 |
|  |  |  |  |  |  |  |
| BMI#German test scores |  |  |  |  |  |  |
| Underweight | 0.048 | 0.103 | 0.470 | 0.641 | -0.154 | 0.249 |
| Overweight | 0.020 | 0.124 | 0.160 | 0.874 | -0.225 | 0.264 |
| Obese | -0.012 | 0.184 | -0.060 | 0.949 | -0.374 | 0.350 |
|  |  |  |  |  |  |  |
| **Mathematics** |  |  |  |  |  |  |
| Underweight | -0.009 | 0.162 | -0.060 | 0.956 | -0.327 | 0.309 |
| Overweight | -0.313 | 0.180 | -1.740 | 0.083 | -0.667 | 0.041 |
| Obese | -0.535 | 0.309 | -1.730 | 0.084 | -1.143 | 0.072 |
|  |  |  |  |  |  |  |
| Mathematics test scores | 0.782 | 0.041 | 19.200 | 0.000 | 0.702 | 0.862 |
|  |  |  |  |  |  |  |
| BMI#Mathematics test scores |  |  |  |  |  |  |
| Underweight | -0.056 | 0.101 | -0.560 | 0.576 | -0.254 | 0.141 |
| Overweight | -0.098 | 0.136 | -0.720 | 0.469 | -0.365 | 0.169 |
| Obese | 0.006 | 0.205 | 0.030 | 0.977 | -0.398 | 0.409 |

Note: The table reports log-odds ratio of the main effects and interaction between BMI and students’ test scores obtained from a hierarchical three-level ordered logit model on German and mathematics test scores. Control variables are common to the main models presented in the article.
